# On the Role of the Interlayer Interactions in Atomistic Simulations of Kaolinite Clay

**DOI:** 10.3390/molecules29194731

**Published:** 2024-10-07

**Authors:** Zoltán Ható, Tamás Kristóf

**Affiliations:** Center for Natural Sciences, University of Pannonia, P.O. Box 1158, H-8210 Veszprém, Hungary; hato.zoltan@mk.uni-pannon.hu

**Keywords:** kaolinite, atomistic simulation, classical force field, layer–layer interaction

## Abstract

A systematic simulation study was performed to investigate the interlayer interactions in a 1:1 layered phyllosilicate clay, kaolinite. Atomistic simulations with classical realistic force fields (INTERFACE and ClayFF) were used to examine the influence of the individual non-bonded interactions on the interlayer binding in the kaolinite model system. By switching off selected pairwise interactions in the applied force fields (leaving the intralayer interactions intact), it was confirmed that the tetrahedral–octahedral-type pairwise interactions held the kaolinite plates together and that interlayer hydrogen bonding, modeled by Coulombic forces, played a dominant role in this. Furthermore, it was observed that the number of hydrogen bonds formed had a significant influence on the basal spacing, and thus there was a striking change in the layer–layer interaction strength when there were only two kaolinite plates in the system, rather than several plates, as in real kaolinite particles. Contrary to expectations, the dispersion forces of the studied force fields alone were found to be strong enough to hold the kaolinite plates together.

## 1. Introduction

Kaolinite belongs to the clay mineral group of phyllosilicates and contains slit pores in the size range of small molecules. The kaolinite particles consist of book-like packets of basal layers, tightly attached to each other by secondary chemical bonds. The basal layers are electroneutral, but the connection of two different constituent sheets gives them some polarity. One sheet is composed of SiO_4_ tetrahedral elements, with a regular hexagonal arrangement, bonded through oxygen atoms to the other sheet of AlO_2_(OH)_4_ elements with an octahedral (dioctahedral) structure. The formula of the C1 symmetry unit cell of the crystal is Al_2_Si_2_O_5_(OH)_4_. A few small polar molecules can be spontaneously incorporated into the space between the layers (intercalation), and thus stable complexes with a basal spacing usually close to 1 nm are formed [1]. For practical reasons, attempts are made to replace the incorporated smaller molecules with larger ones that cannot intercalate in a spontaneous way [2]. Thus, in a series of steps, kaolinite complexes of increasing basal spacing can be formed, and even the fragmentation of the kaolinite particles into smaller assemblies of layers (delamination) or individual layers (exfoliation) can take place [3,4]. The kaolinite complexes created with guest molecules or the curved structures that are eventually formed from individual kaolinite layers (plates) [5,6] can have a variety of applications as additives or fillers, catalyst supports, special purpose adsorbents, and functionalized surface sensors [7].

The most important measurable characteristic of kaolinite–guest molecule systems is the distance between the basal layers [1]. This can also be determined easily by molecular simulations using appropriate atomic interaction models. Simulation reproduction of the experimental layer repeat distances can confirm the reality of the simulations. Both complexes with primary reagents (e.g., various amides) that can be introduced directly into the interlayer space of kaolinite [1,8,9] and complexes with reagents that cannot be introduced in one step [10,11,12,13] have been studied previously. For the primary reagents, atomistic simulation studies have raised the possibility of the existence of two types of stable basal spacings [14]. Among other findings, we showed that kaolinite layers facing the interior of guest molecule-filled pores with their octahedral surfaces interact more strongly with the guest molecules [15].

Materials science researchers are nowadays dealing more intensively with the exfoliation of kaolinite [3,6,16,17]. This makes it worth revisiting in more detail the question of what actually holds the kaolinite layers together in book-like packets. A general and brief explanation is that a multitude of hydrogen bonds forming between the tetrahedral SiO_4_ surface of one kaolinite layer and the octahedral AlO_2_(OH)_4_ surface of the other are responsible for the strong interlayer binding. In atomic-scale modeling, however, we know that the picture is more nuanced: all atom–atom interactions between the layers play a role in this. In the following, we will address this issue at the level of classical atomic interactions using the realistic INTERFACE [18] and ClayFF [19] force fields. At this level of testing, we could not naturally exclude the influence of discretization of the potential energies down to the scale of individual atoms and of the empirical parameterization of the interaction models. Furthermore, this type of force field (including e.g., the ReaxFF force field [20], too) had to be parameterized by taking into account our common-sense chemical knowledge, and thus we can expect that many of the following test results are predictable in advance or can be considered target features in the force field development process. However, the applied force fields are actually general force fields, not specifically developed for kaolinite, and on the other hand, their parameterization was completed on complex experimental datasets. We are not aware of similar studies on the subject, and a systematic investigation of this important issue seems relevant.

## 2. Results and Discussion

In our study, constant pressure and temperature molecular dynamics (NpT-MD) simulations were carried out at 298 K and 101.3 kPa. Two parallel kaolinite layers were placed in the simulation box, where each layer consisted of 48 unit cells with 96 Al, 96 Si, 432 O, and 192 H atoms. The initial atomic positions were set according to the experimental crystal structure [21]. Periodic boundary conditions were applied in all the directions of the crystallographic a, b, and c axes of the kaolinite (the lattice parameters are a = 0.5154 nm, b = 0.8942 nm, c = 0.7391 nm, α = 91.93°, β = 105.05°, and γ = 89.80°). This way, infinite periodic kaolinite layers were modeled in a small simulation box with side lengths in the xy plane between 3 and 4 nm and the system implicitly involved an infinite number of kaolinite layers. This solution is closer to reality compared to the case if we had used small finite-sized layers, because the common experience is that the periodic layers retain their original planar geometry in these simulations. This follows the real behavior of kaolinite particles, where secondary chemical bonds pull the adjacent layers to a proximity of 0.71–0.72 nm and keep the layers in parallel planes. The size of the planar layers in real kaolinite particles is well above 100 × 100 nm. With finite simulation layers, the classical force field model systems give a good approximation to reality only in this colloidal size range [22] (the number ratio of the outer surface atoms to the inner atoms has a fundamental influence on the behavior of the layers). In such a size range, however, the alternative atomic resolution simulations are computationally extremely demanding.

The influence of individual non-bonded interactions on the interlayer binding in the kaolinite model system was tested by switching off the selected pairwise interactions in the applied force fields. In each case, only intermolecular (interlayer) pairwise interactions were selected, leaving the intramolecular (intralayer) ones that fundamentally affect the cohesive strength of these model layers intact. The software package used allows for the selection of individual atom pairs to suppress their interactions. Technically this was carried out by assigning different atom types to the same atoms in different layers. When the tetrahedral or the octahedral sheet of the kaolinite layer was treated separately as a unit, the bridging O atoms connecting these sheets were evenly distributed between the two sheets (see also Table 1 in Section 3).

**Figure 1:**
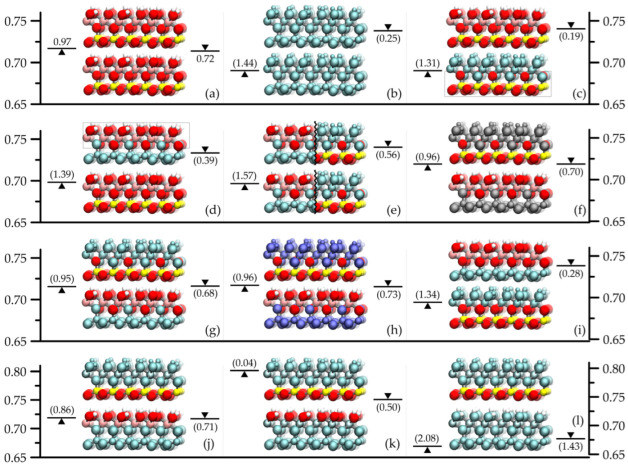
Illustration of the simulation results for the full periodic system with two kaolinite layers obtained by switching off selected pairwise interactions. The CPK color convention is applied to atoms for which all original interactions are preserved (Al, Si, O and H atoms are pink, yellow, red, and white, respectively). The atoms for which all interlayer interactions have been switched off are gray, and those for which only the interlayer Lennard-Jones or Coulombic interactions have been retained are cyan or blue, respectively (color modifications are not depicted for the periodic interactions). The atoms shown in the frame are considered to be interchangeable atom types between the two layers when calculating the interlayer interactions across the illustrated interlayer space. The values of the calculated basal spacing are indicated by the positions of the symbols 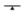 and 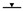 with respect to the y-axis scale for the INTERFACE and ClayFF force fields, respectively (values are in nm). Below or above the basal spacing symbol is the number of hydrogen bonds per surface hydrogen atom.

To attain good agreement with the experimental basal spacing results (~0.72 nm) in the simulated pure and unmodified kaolinite system, it was essential to treat the long-range part of the Coulombic interactions of both force fields by the Ewald summation [23]. This fact is known from the literature [14,22] and was used for preliminary testing of the force fields and the simulation setup and methodology (see Figure 1a). For further control experiments and simulation data, the reader is referred to our earlier published works and studies of various authors in the reference list.

**Figure 2:**
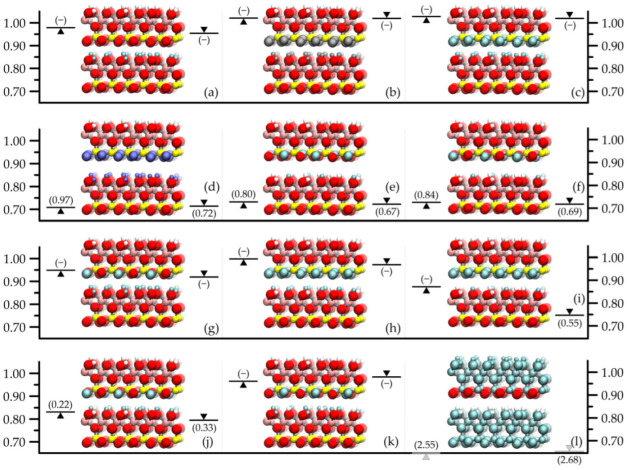
Same as Figure 1.

Figure 1 and Figure 2 summarize the main simulation results obtained in this work and calculated by retaining the bonded and non-bonded interactions within the layers. As a first step (and as a further check), we eliminated all pairwise interactions between the layers and ended up with the expected indeterminate systems with both force fields such that any basal spacing was possible, even layer–layer overlapping configurations. When we switched off only the charge–charge interactions between the layers (Figure 1b), the remaining Lennard-Jones atom–atom repulsions and attractions resulted in basal spacings broadly similar to the original ones. However, here the different parametrization of the two force fields is already apparent: without charge–charge attractions and repulsions, the average layer repeat distance shifted by approximately −0.03 nm and +0.02 nm for the INTERFACE and ClayFF force fields, respectively. The increase occurred despite the fact that there is no Lennard-Jones potential term on the H atoms in the ClayFF force field, and therefore the H atoms could in principle get closer to the other layer surface. This behavior was practically repeated in the artificial cases where the charge–charge interactions of atoms of either the octahedral or the tetrahedral sheet with atoms of the opposite or both sheets of the neighboring layer were switched off (Figure 1c–e). At the same time, the removal of octahedral–octahedral- and tetrahedral–tetrahedral-type Coulombic (Figure 1g) or Lennard-Jones interactions (Figure 1h) caused negligible shifts in the basal spacings from the original values (see Figure 1f, which shows the combination of them). All these results are consistent with the expectation that the tetrahedral–octahedral-type pairwise interactions are predominantly responsible for holding the kaolinite layers/plates together. It should be noted that even with such relatively high degrees of interaction removal, we did not observe any major changes in the structure of the individual infinite layers. This means that the applied force fields (especially the ClayFF force field, where there is hardly any bonded interaction within the layers) were correctly parameterized in the sense that the stability of the layers does not essentially require the influence of the neighboring layers. All of the above manipulations caused negligible changes in the calculated average layer–layer potential energy for the INTERFACE force field when the basal spacing remained the same, and in the other cases, the observed negative shifts of up to 20% in this energy reflected the decrease in basal spacing rather than the removal of certain interactions. The behavior of the ClayFF force field was quite different in this respect. The calculated average layer–layer potential energy, originally strongly negative (see later, in Figure 4), five times the value of the other force field, dropped to the energy level of the other force field when the interaction removal included the tetrahedral–octahedral-type pairwise interactions and showed only 5–10% positive changes when the tetrahedral–octahedral-type pairwise interactions were retained in the interaction removal. One of the main reasons for these differences is that the ClayFF force field atoms are more ionic in nature (they have much larger point charges). The second reason is also a matter of principle in the sense that the net charge of either the octahedral or the tetrahedral sheet in the ClayFF force field is zero. The net charge in the different sheets is unbalanced in the INTERFACE force field, even with and without the bridge atoms.

The main problem can be further investigated through the decomposition of the sheets into smaller units. The tetrahedral sheet can naturally be represented by the tetrahedral Si and O atoms without bridging O atoms, and the octahedral sheet can be treated in the same way (i.e., without bridging O atoms and any associated H atoms). Removing the electrostatic attractions or repulsions between the atoms of the SiO and AlOH groups of the facing kaolinite plate surfaces (Figure 1i) had practically the same effect as removing all charge–charge interactions between the kaolinite plates (cf. Figure 1b). Conversely, if we allowed electrostatic interactions merely between these groups and switched off all other electrostatic interlayer interactions (Figure 1j), we obtained the same results as when we switched off the octahedral–octahedral and tetrahedral–tetrahedral-type atomic interactions (cf. Figure 1g). This simply means that the contribution of the bridge O atoms or any associated H atoms to the interlayer attraction or repulsion is not particularly important. It logically followed that when only the OH groups of the surface AlOH groups were considered in the latter case (Figure 1k), the calculated basal spacings had increased due to increased repulsion. However, considering here the H atoms of the AlOH groups alone (Figure 1l), a notable reduction in the original basal spacings occurred. This fact draws attention to the importance of hydrogen bonds between the layers. We therefore chose to focus on the interlayer O-H interactions, as follows.

**Figure 3:**
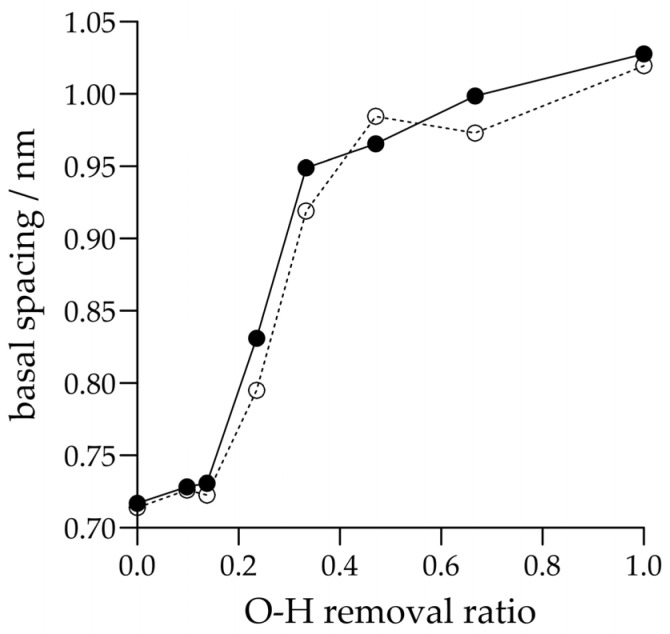
Basal spacing for the INTERFACE (full line and full symbol) and ClayFF (dotted line and open symbol) force fields as a function of the removal ratio of the surface–surface O-H Coulombic interactions in the simulation (the exact removal ratios were determined from the atom–atom coordination numbers calculated from the corresponding radial distribution functions of the original crystal structure at a distance of 0.33 nm). Statistical uncertainties do not exceed the symbol size.

In the investigation of the hydrogen bonding, the first artificial case was the temporary elimination of all the interlayer Coulombic interactions involving surface H atoms (Figure 2a). The basal spacing then showed a significant increase with both force fields up to values between 0.9 and 1.0 nm. When only the surface–surface O-H Coulombic interactions were removed (Figure 2c, cf. Figure 2b), these increases became even larger, exceeding 1.0 nm. It is worth noting that if the same was done with the Lennard-Jones interactions (Figure 2d) for the INTERFACE force field, the basal spacing barely changed compared to the origin (this modification is irrelevant for the ClayFF force field, because there is no Lennard-Jones potential on its H atoms). Thus, it is clear that in these classical models, hydrogen bonding is naturally dominated by electrostatic interactions. Since the global strength of hydrogen bonding between the kaolinite model plates depends also on the number of possible close O-H contacts (in addition to the partial charge parameters of the corresponding O and H atoms of the force fields), we also tried to vary the number of O-H contacts. In the crystallographic information file of the experimental crystal structure of kaolinite [21], there are three types of surface hydroxide O atoms (marked as O3, O4 and O5) and three types of surface H atoms (marked as H2, H3 and H4) in equal proportions (see also Table 1 in Section 3). Thus, we were able to reduce the number of possible O-H contacts by a third if we omitted one of the three types of H atoms or one of the three types of O atoms. By taking two types of them, the reduction ratio became two-thirds and by taking two types of both of them, the reduction ratio became one-ninth. In this way, by keeping all the interlayer interactions except the O4-H4 or O5-H4 (or, e.g., O3-H2) pairwise electrostatic interactions (Figure 2e,f), the circa one-ninth decrease in the number of possible O-H contacts was already reflected in a slight increase in the layer spacing (about 0.01 nm). By removing, e.g., the O5-H2, O5-H3, and O5-H4 pairwise electrostatic interactions (Figure 2g), the increase was larger (more than 0.2 nm) and by removing, e.g., the O3-H3, O4-H3 and O5-H3 as well as O3-H4, O4-H4 and O5-H4 pairwise electrostatic interactions (Figure 2h), there was a further increase. We checked whether the change in basal spacing was really gradual by using intermediate settings (see Figure 3): we also tested the cases of O3-H4, O4-H4, and O5-H4 removals, O5-H3 and O5-H4 removals, and O3-H2, O4-H2, O3-H3, and O4-H3 removals (Figure 2i–k). In the above circumstances, the average layer–layer potential energy was found to be moderately negative, usually well below, in absolute terms, the values of the unmodified systems, but these changes practically reflect the increase in basal spacing. In the above, the number of *possible* hydrogen bonds is mentioned. Figure 1 and Figure 2 show the average number of resulting non-bonded O-H distances per surface H atom in the simulations that meet the hydrogen bonding criterion (for artificial cases, these numbers are in parentheses). These numerical data on the amount of hydrogen bonds can also provide interesting chemical insights from a macroscopic perspective. Regarding the data on the original crystal structure, which approach 1 from below, the lower value for the ClayFF force field (see Figure 1a) indirectly indicates that the dispersion interactions have a significant effect (c.f. with the basal spacing difference in Figure 1b), even if this is sometimes hidden behind the dominant Coulombic interactions (both force fields were parameterized using the lattice treatment of the electrostatic interactions, which magnifies the influence of these interactions [24]). Overall, however, it can be seen that the number of hydrogen bonds is broadly in line with the variation in the basal spacing: they can even be significantly higher than 1 for spacings smaller than those of the original crystal structure, and become zero for spacings approaching 0.9 nm or more.

As a final test of the significance of hydrogen bonding, we switched off all the electrostatic interactions between the kaolinite layers except for the surface–surface O-H interactions (Figure 2l), thus eliminating the possible net Coulombic repulsion between the layers that would occur without these O-H pairs. As a result, the pure O-H attraction brought the layers as close together as the Lennard-Jones interactions allowed (0.65 and 0.66 nm for the INTERFACE and ClayFF force field, respectively), and this was accompanied by extremely large negative layer–layer potential energies: the systems reached the edge of the so-called Coulomb catastrophe.

In many cases, we were able to forecast the simulation results from the potential energy profiles (total potential energy of the system and layer–layer interaction energy) calculated for the original crystal structure [21] but with varying layer–layer distances. However, the location of the energy minimum of the layer–layer interaction energy profile is only an approximation of the simulated basal spacing, since in this calculation, the atomic positions within the layers correspond to ideal crystallographic positions: the differences were found to be larger for the ClayFF force field, where there are few bonded interactions between the neighboring atoms in the layer (the discrepancy can also be larger when the calculated energy curve is flat around the energy minimum). An alternative way of making such a prediction would have been to calculate the free energy profile between the plates [25].

**Figure 4:**
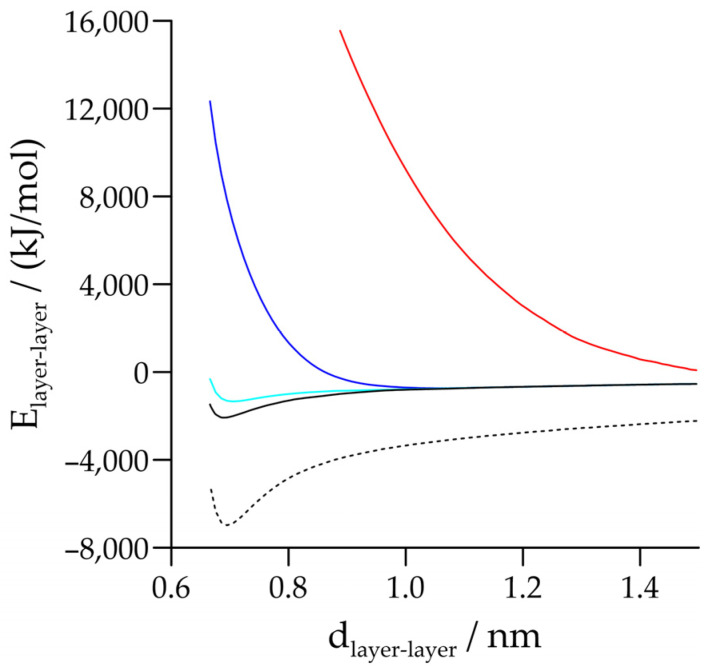
Layer–layer interaction energy (E_layer–layer_) calculated with the original crystal structure for the INTERFACE (full line) and ClayFF (dotted line) force fields as a function of the layer–layer distance. Line in cyan stands for the case, where only the Lennard-Jones atom–atom interactions were retained from the interlayer interactions. The blue and red lines represent the energy profiles obtained by the removal of the surface–surface O-H Coulombic interactions in the normal periodic and in the quasi-non-periodic systems, respectively.

Figure 4 shows the layer–layer interaction energy profiles for the original crystal structure with varied basal spacings, illustrating the differences between the two unmodified force fields. This figure also includes data for the case, where only the Lennard-Jones atom–atom interactions were retained from the interlayer interactions (note that the overall shape is very similar to that obtained for the unmodified case, but the energies are higher). The most striking result of the potential energy profile calculations was obtained when the electrostatic interactions of the tetrahedral–octahedral type were generally excluded or, within this category, when only the surface–surface O-H interactions were excluded. In these cases, we observed qualitative differences between the normal periodic system and that with a large and constant box size in the z direction. In the latter system, the layer–layer interaction energy curves did not show a negative minimum as in the normal periodic system, but decreased continuously, mostly with positive values, over the whole distance range. This suggests that in contrast to the periodic cases, the residual interactions are no longer sufficient to hold the kaolinite layers together. Note that the system with a large and constant box size in the z direction is quasi-non-periodic in the direction perpendicular to the kaolinite sheets. Consequently, it contains only two truly interacting kaolinite layers, and the formation of hydrogen bonds is possible with one pair of facing layer surfaces.

**Figure 5:**
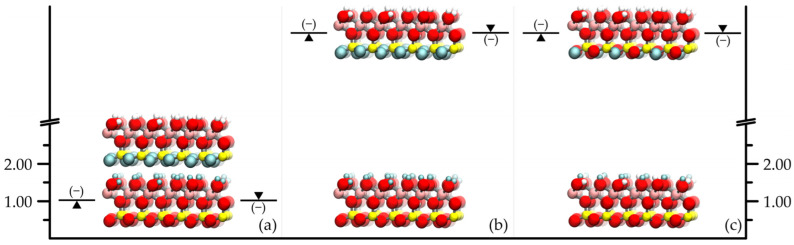
Illustration of the simulation results for the full periodic (**a**) and the quasi-non-periodic (**b**,**c**) systems with two kaolinite layers obtained by switching off all the surface–surface O-H (**a**,**b**) and the O5-H3 plus O5-H4 (**c**) Coulombic interactions. All other specifications are the same as in Figure 1.

To clarify the latter issue, most of the NpT-MD simulations were repeated with the quasi-non-periodic system. Here, the simulation box size in the z direction was kept at a constant value (the size change in the z direction was switched off), which was at least three times the possible maximum cut-off radius. We found that the quasi-non-periodic simulations gave similar results to the full-periodic simulations for the pure and unmodified kaolinite system, except that the experiments were slightly underestimated with the INTERFACE force field (by ~0.01 nm). As expected, the applied pressure of 101.3 kPa bar was sufficiently low such that the effect of switching off the pressure control in the z direction was negligible. In many cases, we observed that there were at least qualitative agreements between the results of the two system variants. However, according to the forecast of the calculated energy profiles, in the quasi-non-periodic system, the kaolinite plates tried to be as far apart as possible with both force fields when the tetrahedral–octahedral type (surface–surface) O-H electrostatic interactions were switched off (as an illustration, see Figure 5). This means that the residual net Lennard-Jones energy and the electrostatic interactions of the tetrahedral–tetrahedral and octahedral–octahedral types could no longer hold *two* kaolinite layers together. Interestingly, this happened even when only a few types of surface–surface O-H electrostatic interactions were eliminated.

**Figure 6:**
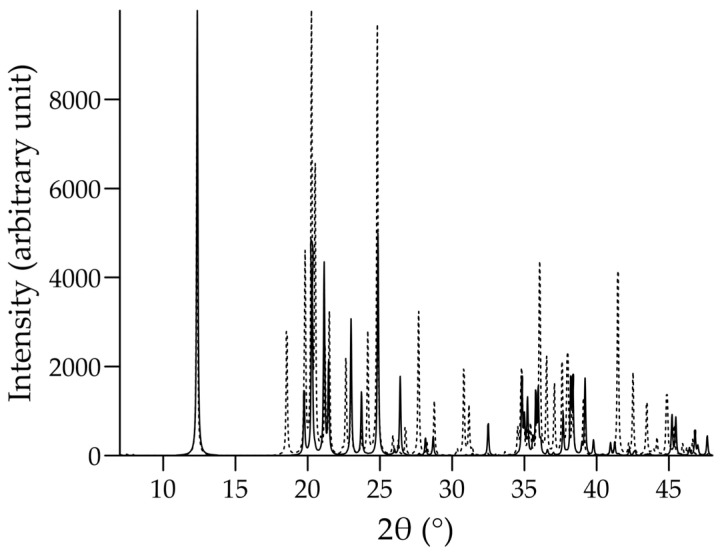
XRD patterns calculated (with X-ray wavelength of 0.154056 nm and step size of 0.02° 2θ) for the simulated pure and unmodified kaolinite system with the INTERFACE force field (full line) and for the same system without using the charge–charge interactions between the layers (dotted line). For the modified system, the XRD pattern was calculated after applying an artificial correction: one of the layers was shifted along the actual crystallographic c axis to eliminate the effect of the slightly different basal spacings of the two systems (see the identical position of the primary indicator peaks of the basal spacing around 2θ ≅ 12.3°).

Overall, our investigations proved the dominant role of Coulombic interactions in the properties of kaolinite. However, there is an apparent contradiction in that the simulations with interlayer Lennard-Jones atom–atom interactions alone (without interlayer charge–charge attractions and repulsions) resulted in basal spacings similar to the original ones. Returning to this question, we calculated the X-ray diffraction (XRD) patterns of the clay structures obtained from simulations to highlight possible structural differences that are not really visible in the examined simulation snapshots. For this, Free Mercury software (version 3.7) was used [26]. We considered the XRD patterns as fingerprints and compared the calculated patterns of the simulated pure and unmodified kaolinite system with that of the experimental structure [21]. Reasonably good agreement was found between the experimental structure and that obtained with the INTERFACE force field. The agreement was slightly weaker when the ClayFF force field was applied in the simulation. We also observed that very similar patterns can be produced by removing the possible interlayer Lennard-Jones atom–atom interactions, but retaining all the Coulombic ones (to prevent interlayer overlaps, at least the interlayer Lennard-Jones interactions between the SiO and surface OH groups had to be retained). The comparison of the XRD pattern calculated for the simulated pure and unmodified kaolinite system with the INTERFACE force field and that of the same system without using the charge–charge interactions between the layers (keeping the interlayer Lennard-Jones atom–atom interactions alone) is shown in Figure 6. The difference is striking between the two patterns. There are more reflecting planes in the structure obtained by Coulombic interaction removal. The XRD pattern of the modified system looks more complex, having more intensive peaks in the higher 2θ region. Thus, the interlayer Coulombic interactions are apparently necessary to preserve the finer details of the original crystal structure. Presumably, these charge–charge interactions are also necessary to preserve the *planar* layer structure in the book-like kaolinite particles. This issue, however, requires further investigation.

## 3. Methods

For the interatomic potentials, two widely accepted classical all-atom force fields, INTERFACE [18] and ClayFF [19], were used (see Table 1), which are suitable for atomic-level modeling of minerals and mineral interfaces. These fully flexible types of force fields contain quadratic bond stretching, angle bending, and non-bonded Coulombic and 12-6 Lennard-Jones energy terms (the non-bonded intramolecular interactions are only applied to atom pairs separated by at least three bonds). Except for the O-H pairs, the ClayFF force field considers all possible atom–atom pairs as being non-bonded. For the unlike Lennard-Jones interactions, the Lorentz–Berthelot combination rule was used. The charge–charge interactions were treated by the Ewald summation [23]. In the simulations, the cut-off radius for both the charge–charge and Lennard-Jones interactions was always near but below half of the smallest box side length. If the net charge of the system was non-zero after switching off the selected pairwise interactions in the applied force fields, it was neutralized by a uniform background plasma, and a corresponding correction was applied to the energy to reduce the unwanted side effect of the artificially created subsystem [27].

The NpT-MD runs were performed using the LAMMPS simulation package (v. 2 Aug 2023—Update 3) [28], where the velocity-Verlet integrator was applied with a time step of 1 fs and the temperature and pressure were controlled with the Nosé–Hoover type thermostat and barostat [29]. Deactivation of the selected pairwise interactions was realized by using a soft-core modified Coulomb + Lennard-Jones pair potential containing an activation parameter λ (when λ = 0, the corresponding pair interaction is deactivated). The number of hydrogen bonds formed in the simulations was determined from the number of neighboring atoms calculated from radial distribution functions for non-bonded O-H pairs that are within a distance of 0.26 nm (then, mainly because of the parallel alignment of the two kaolinite layers, the usual O-H-O angle and O-O distance criteria are automatically satisfied). Besides molecular simulation, additional energy profile calculations were carried out for the original crystal structure [21], except that the basal spacing was artificially adjusted.

## 4. Conclusions

A systematic simulation study was carried out to answer the question of what actually holds kaolinite layers together. The question posed has been investigated at the level of classical realistic force fields. The classical force fields are of course approximate interaction models, but this was somewhat compensated by performing the same manipulations for two widely accepted but considerably different force fields, the INTERFACE and ClayFF force fields. As these interaction models were parameterized in dissimilar ways, our analysis of the possible deviations in their behavior focused on empirical evidence rather than on possible theoretical explanations for the differences. At the same time, however, the mutually reinforcing results of the INTERFACE and ClayFF force fields make our picture of the real behavior of kaolinite more plausible.

Our main results can be summarized very briefly. (1) We have confirmed that the tetrahedral–octahedral type pairwise interactions are responsible for holding the kaolinite plates together. (2) Hydrogen bonding does indeed play a prominent role in this. (3) The number of hydrogen bonds formed has a significant influence on the basal spacing. (4) The extent of hydrogen bonding per plate is halved when there are only two kaolinite plates interacting, rather than several plates in a multi-element kaolinite particle, and this will make it much easier to break the connection between two kaolinite plates. (5) Finally, and interestingly, we found that the dispersion forces of the studied force fields alone are strong enough to hold the kaolinite plates together (with a slightly disturbed layer structure).

From a broader perspective of the kaolinite intercalation complexes, we can draw the following conclusions. Earlier simulation studies performed by us and other authors have raised the possibility of the existence of two types of stable basal spacing in kaolinite-primary intercalation reagent systems [1,8,9], but mostly the smaller one is observed in experiments. To resolve the discrepancy with the experimental results, it was assumed that there is in reality a mostly unbridgeable energy gap between the two states with different basal spacings. The results of our present study do not contradict the experimental explanation for the formation of the smaller basal spacing (between 1.0 and 1.1 nm): the hydrogen bonds between the layers are broken by the primary intercalation reagent molecules and the layers are interconnected by these molecules themselves, properly oriented within the interlayer space to form as many hydrogen bonds or secondary chemical bonds with the layer atoms as possible. As we did not find basal spacings well above 1.1 nm, the reasons for the formation of the other possible stable basal spacing (around 1.4–1.5 nm) cannot be explained by the properties of the kaolinite plates themselves (e.g., by the properties obtained from the present calculations for the variation in the interactions between the kaolinite layers as a function of their distance). We assume that this requires the formation of two parallel layers of intercalation reagent molecules between the kaolinite plates and the presence of strong intermolecular interactions between these layers. All the results of our simulation studies to date point in this direction.

## Figures and Tables

**Table 1:** Parameters of the applied force fields.

INTERFACE Non-Bonded Interaction Parameters				
Sheet	Atom Type	q/e	R_0_/nm *	ε/(kJ/mol)
**octahedral sheet**	Al1, Al2	+1.45	0.42	0.20934
	Oh2, Oh3, Oh4	−0.68333	0.35	0.10467
	H2, H3, H4	+0.2	0.1098	0.0544284
	O2 (bridging)	−0.75833	0.35	0.10467
	Oh1 (inner/bridging)	−0.68333	0.35	0.10467
	H1 (inner/bridging)	+0.2	0.1098	0.0544284
**tetrahedral sheet**	Si1, Si2	+1.1	0.4	0.20934
	O3, O4, O5	−0.55	0.35	0.10467
	O1 (bridging)	−0.75833	0.35	0.10467
**INTERFACE Bonded Interaction Parameters**				
**Bonds**	**r_0_**			**K_r_/(kJ(mol·nm^2^)^−1^)**
Si-O, Al-O bonds	1.05·Exp. **			360,064.8
O-H bonds	1.05·Exp. **			41,4493.2
**Angles**	**Θ_0_**			**K_Θ_/(kJ(mol·rad^2^)^−1^)**
O-Si-O, O-Al-O, Al-O-Si angles	Exp. **			1423.512
Al-O-H angles	Exp. **			96.2964
**ClayFF Non-Bonded Interaction Parameters**				
**Sheet**	**Atom Type**	**q/e**	**R_0_/nm** *	**ε/(kJ/mol)**
**octahedral sheet**	Al1, Al2	+1.575	0.4794	5.56388 × 10^−6^
	Oh2, Oh3, Oh4	−0.95	0.3553	0.65017
	H2, H3, H4	+0.425	–	–
	O2 (bridging)	−1.05	0.3553	0.65017
	Oh1 (inner/bridging)	−0.95	0.3553	0.65017
	H1 (inner/bridging)	+0.425	–	–
**tetrahedral sheet**	Si1, Si2	+2.1	0.3706	7.70065 × 10^−6^
	O3, O4, O5	−1.05	0.3553	0. 65017
	O1 (bridging)	−1.05	0.3553	0. 65017
**ClayFF Bonded Interaction Parameters**				
**Bonds**	**r_0_/nm**			**K_r_/(kJ(mol·nm^2^)^−1^)**
O-H bonds	0.1			463,700
**Angles**	**Θ_0_/deg**			**K_Θ_/(kJ(mol·rad^2^)^−1^)**
Al-O-H angles	109.47			251.04

* The usual Lennard-Jones size parameter σ is equal to R_0_/2^1/6^. ** Exp. denotes the experimental distance and angle values [21].

## Data Availability

Dataset available on request from the authors.
